# The Antimicrobial Peptide Gramicidin S Enhances Membrane Adsorption and Ion Pore Formation Potency of Chemotherapy Drugs in Lipid Bilayers

**DOI:** 10.3390/membranes11040247

**Published:** 2021-03-30

**Authors:** Md. Ashrafuzzaman

**Affiliations:** Biochemistry Department, Science College, King Saud University, Riyadh 11451, Saudi Arabia; mashrafuzzaman@ksu.edu.sa

**Keywords:** chemotherapy drug, antimicrobial peptide, membrane, ion pore

## Abstract

We recently published two novel findings where we found the chemotherapy drugs (CDs) thiocolchicoside (TCC) and taxol to induce toroidal type ion pores and the antimicrobial peptide gramicidin S (GS) to induce transient defects in model membranes. Both CD pores and GS defects were induced under the influence of an applied transmembrane potential (≈100 mV), which was inspected using the electrophysiology record of membrane currents (ERMCs). In this article, I address the regulation of the membrane adsorption and pore formation of CDs due to GS-induced possible alterations of lipid bilayer physical properties. In ERMCs, low micromolar (≥1 μM) GS concentrations in the aqueous phase were found to cause an induction of defects in lipid bilayers, but nanomolar (nM) concentration GS did nothing. For the binary presence of CDs and GS in the membrane-bathing aqueous phase, the TCC pore formation potency is found to increase considerably due to nM concentration GS in buffer. This novel result resembles our recently reported finding that due to the binary aqueous presence of two AMPs (gramicidin A or alamethicin and GS), the pore or defect-forming potency of either AMP increases considerably. To reveal the underlying molecular mechanisms, the influence of GS (0–400 nM) on the quantitative liposome (membrane) adsorption of CD molecules, colchicine and TCC, was tested. I used the recently patented direct detection method, which helps detect the membrane active agents directly at the membrane in the mole fraction relative to its concentrations in aqueous phase. We find that GS, at concentrations known to do nothing to the lipid bilayer electrical barrier properties in ERMCs, increases the membrane adsorption (membrane uptake) of CDs considerably. This phenomenological finding along with the GS effects on CD-induced membrane conductance increase helps predict an important conclusion. The binary presence of AMPs alongside CDs in the lipid membrane vicinity may work toward enhancing the physical adsorption and pore formation potency of CDs in lipid bilayers. This may help understand why CDs cause considerable cytotoxicity.

## 1. Introduction

**Figure 1:**
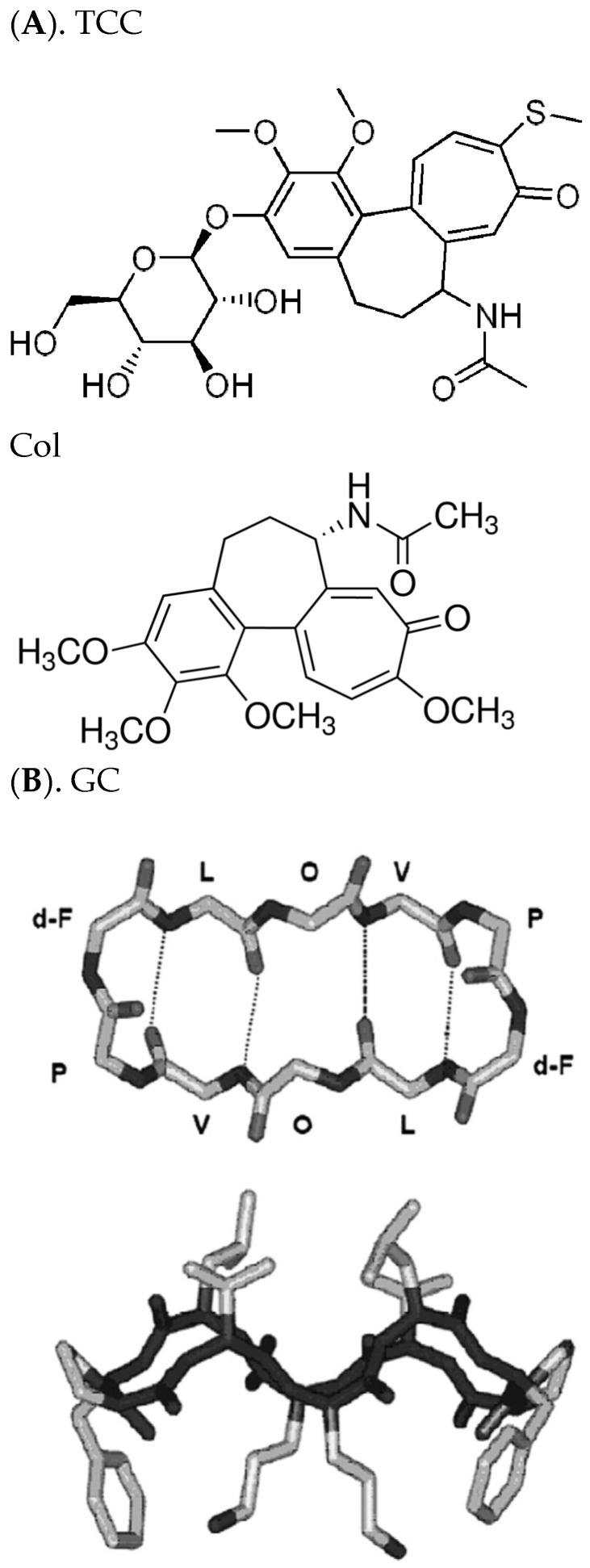
(**A**). Thiocolchicoside (TCC) structure [1,2] and Col structure (see Sigma, www.sigmaaldrich.com accessed on 19 February 2021). (**B**). Gramicidin S (GS) structures [9]. The top panel (**B**) shows a three-dimensional (3D) view of the GS molecule perpendicular to the plane of the ring, which illustrates the peptide backbone structure and the positions of the hydrogen bonds in the antiparallel β-sheet region. The bottom panel shows a view in the plane of the ring, which indicates the spatial disposition of the hydrophobic Val and Leu residues (top) and the basic Orn residues (bottom) relative to the peptide ring.

Chemotherapy drugs (CDs) thiocolchicoside (TCC) and taxol have been found to induce ion pores inside lipid bilayer membranes [1,2]. These pores are predicted to be toroidal type [3,4]. A toroidal type pore, e.g., induced by TCC, which is a derivative of parent compound colchicine (Col) (Figure 1A), generally requires two lipid monolayers to meet each other across the pore opening. A group of versatile ion pores or channels, such as β-helical, toroidal, barrel-stave pores, etc., are induced in lipid membranes by various antimicrobial peptides (AMPs) [3,4,5,6,7,8]. In addition, a cationic cyclic decapeptide gramicidin S (GS) (Figure 1B) is known to induce defects in lipid membranes [9]. All of these induced pores or channels and defects ultimately disrupt the lipid bilayer’s electrical insulation properties and force the membrane to conduct currents instantaneously or over a period of time. Below the membrane electrical barrier property disrupting concentrations, some of these agents may still alter general bilayer physical properties. In this article, I shall present experimental results and convincing analysis on underlying molecular mechanisms suggesting that GS below its defect-forming concentrations may regulate the CD pore-forming potency in lipid bilayers. 

Although two CD molecules (TCC and taxol) [1,2] and the GS [9] are found to compromise bilayer electrical barrier properties in electrophysiology (EP) record experiments, both of these structurally and chemically different agents offer an independent type of membrane actions. Their cellular activities are also distinguishable, but many of these actions are somehow linked to membrane bilayers. The CD candidate Col is primarily used in immune-system diseases [10,11]. It is found to inhibit leukocyte–endothelial cell adhesion [12,13] and the T-cells activation [14] through binding with tubulin dimers, thus preventing the polymerization into microtubules (MTs) [15]. Due to mainly an increased mitosis rate, cancer cells are found to be more vulnerable to Col poisoning relative to normal cells. Col is known to shift the dynamic equilibrium in microtubules (MTs) toward the disassembly phases by sequestering most of tubulin [16] and inducing the slow disassembly of MTs [17]. This is predicted to account for gradual changes in the membrane action potential (decreasing trend) or threshold (increasing trend). The resting potential is altered modestly at Col concentrations ≈2.5 mM. At relatively higher Col concentrations (≈10 mM), the resting potential reduces by as much as up to ≈5 mV [16]. Col is also found to bind with the nuclear periphery and causes disordering in the phospholipid bilayers of the nuclear membrane [18].

GS (see Figure 1B), an amphiphilic molecule, shows to have two charged and somewhat polar Orn, and two d-Phe side chains that project from one molecular side and four hydrophobic Val and Leu side chains that project from the other side [19,20]. This type of conformation is observed to be maintained in water, protic, and aprotic organic solvents with varying polarity, as well as detergent micelles and especially phospholipid bilayers. The GS molecule may undergo a structural change from a more compact type to a relatively extended β-sheet, β-turn conformation as it binds to the phospholipid vesicles [21]. GS is suggested to target the bacterial or eukaryote lipid bilayer membranes; thus, it probably kills bacteria via the permeabilization of their inner membranes [22,23,24,25,26]. GS and a few analogs have been found to permeabilize model membrane lipid bilayers [9,24]. Differential scanning calorimetry (DSC) studies suggest that GS perturbs phospholipid bilayer thermotropic phases, offering more strong perturbations of the anionic as compared to the zwitterionic phospholipid bilayer membrane phases [25]. In the sensitometry and the sound velocity studies, GS is found to decrease the host phospholipid bilayer density and volume compressibility by raising the conformational disorder and the motional freedom of the hydrocarbon chains of the phospholipids [27]. In ^31^P nuclear magnetic resonance (NMR) [28] and X-ray diffraction [29] studies, GS at lower concentrations is found to cause phospholipid bilayer thinning and at high concentrations to favor forming the inverted, nonlamellar cubic phospholipid phases. X-ray studies suggested that the membrane adsorption of GS is found to potentiate the inverted cubic phase formation through increasing negative curvature stress in the bilayer [29]. In Fourier transform infrared (FTIR) spectroscopy studies, GS is found located in the phospholipid bilayer polar/apolar interfacial region near the glycerol backbone region of the lipid molecule, and it penetrates deep into the anionic and relatively more fluid lipid bilayers [30]. However, the depth of GS penetration into this interfacial region can vary somewhat depending on the structure and charge of the lipid molecule. In general, GS associates most strongly with and penetrates most deeply into more disordered bilayers with a negative surface charge. EP experiments suggest that GS at specific concentrations can disrupt the lipid bilayer electrical barrier properties [9,31]. In these two studies, it is suggested that considerably high GS concentrations and transmembrane voltages are needed to let the bilayer conduct ionic currents. We were first to predict the GS effects on lipid bilayers as nothing but to induce transiently appearing defects, unlike substantially stable ion pores/channels [9]. All of these studies demonstrate altogether that GS, at various concentrations and physiological conditions, has substantial roles to play in regulating lipid bilayer membrane properties (mostly physical in nature) through often actually getting adsorbed in the membrane.

Both CD and GS are distinguishably active against bilayer barrier properties besides influencing other physiologically relevant properties and parameters of membranes. CD pores break the membrane’s barrier properties by conducting currents across the bilayer over a duration of considerable time [1,2], while GS defects force the membrane to transiently compromise its barrier properties with the current events showing no stability [9]. The time difference between the appearance and disappearance of current events representing GS defects is almost 0 s or undetectable using conventional EP record methods. The induction of GS defects requires a disruption of physical integrity of membrane lipid compositions. The membrane adsorption of GS is expected to generally cause alterations in physical properties even though that might not be considerably enough to induce defects. We could demonstrate any such GS effects on membrane by inspecting the subsequent possible effects on the functions of any integral ion pores/channels. Since we already found that due to the binary presence of two AMPs (gramicidin A (gA) or alamethicin (Alm) and GS) in aqueous phase, the pore or defect-forming potency of either AMP increases considerably over the potency due to its independent presence [32]. GS, at concentration (c_i, nc_) below its membrane conducting concentration (c_i,c_), considerably increases the gA or Alm pore function [32]; see supp. Figure 1 (details to be published by Ashrafuzzaman and Andersen). Therefore, GS is predicted to somehow alter the general membrane properties, which subsequently regulates other AMPs’ pore-forming potencies. GS effects are found to be consistent with a large number of membrane active agents (MAAs) amphiphiles and amphipathic molecules, and other drugs that have been reported to increase the values of both stability τ_ch_ and appearance frequency f_ch_ for β-helical gA channels [33,34,35,36,37]. We have also found substantial effects of amphiphiles triton X-100 (TX100) and capsaicin (Cpsn) on the stability of the appearance frequency of barrel-stave structure forming Alm channels [38], the details of which appear elsewhere by Ashrafuzzaman and Andersen. It is predicted that the aqueous phase dissolved MAAs migrate to the lipid membrane, get physically adsorbed there, and alter bilayer physical properties, perhaps “the membrane stiffness”, by changing the bilayer elasticity, curvature profiles, etc. The migration of both types of MAAs, the specific pore-forming agents (PFAs) and general membrane physical property-regulating agent, to the host membrane may be quantified utilizing our recently developed “direct detection method (DDM)” [39,40]. DDM helps quantify the actual amount of an MAA adsorbed by the membrane. Both types of MAAs may be dissolved concomitantly inside the membrane bathing aqueous phase. Then, we may quantify the relative molar concentrations of the PFAs adsorbed in bilayers in the presence of other MAAs, which may have influences on the membrane physical properties. If the second agent has any effects on the membrane physical properties, that might influence PFA’s membrane adsorption. DDM can address the regulation of such a membrane adsorption process. The altered (reduced) membrane stiffness is predicted to favor the integral channel functions [33,34,35,36]. Although amphiphiles Cpsn and TX100 are known to promote negative and positive curvature profiles of the adsorbing lipid bilayer membrane, respectively [33], both are found to generally stabilize integral ion channels by perhaps mainly reducing the bilayer stiffness [33,36,38]. GS, a negative curvature promoting AMP [29], is also found to stabilize gA and Alm channels in the lipid bilayer, perhaps by also reducing the bilayer stiffness [32] (details yet to be published). The effects of peptide GS (below its membrane-conducting concentrations) on lipid membrane properties may appear universal, which might translate into inducing subsequent effects on integral CD pore functions [1,2]. Thus, this study may open up new avenues on addressing the ways to regulate the CD effects on specific cell membrane properties and general cytotoxicity (unwanted damage to normal cells caused by anticancer drugs), which is one of the major problems of chemotherapy [41,42]. 

## 2. Materials and Methods

### 2.1. Electrophysiology Experiments

A lipid cocktail of phosphoethanolamine (POPE)/phosphatydyleserine (POPS)/phosphatidylcholine (POPC) (5:3:2, *v*/*v*/*v*)/n-decane was used to form planar lipid bilayers over a 150 µm septum of a bilayer cuvette. The cuvette volume was 1 mL, and it was filled with a buffer (0.5 M NaCl, 10 mM HEPES, pH 7.4) in both chambers, the *cis* (recording electrode) and *trans* (reference electrode) sides. 

Col (from Sigma, St. Louis, MO, USA) and TCC (from ChemRoutes, Edmonton, AB, Canada, a gift from Prof. Tuszynski of University of Alberta) stocks were prepared in dimethylsulfoxide (DMSO) (from Burdick and Jackson, Muskegon, MI, USA) (4 mg/mL or 7.1 mM), diluted in buffer for further use (1 mg/mL). GS (from Sigma) stock (200 μM) was prepared using DMSO.

After we had formed the lipid bilayer, we usually waited for 1 h; then, we tested and confirmed the bilayer stability by applying a pretty high transmembrane potential, V = 400 mV. Following a vigorous vortexing, an aliquot of TCC stock was added to the cis chamber buffer while stirring so that we could avoid any drug solubility issues. Twenty min after the addition of TCC, we started recording currents. The current traces across the membrane were recorded at a reasonable transbilayer applied voltage (V) for a considerable amount of time (up to around a min). The membrane current shows occasional nonzero values, representing the conductance events, due to the formation of ion pores in the lipid bilayer. Then, I added an aliquot of GS stock in chambers on both sides of the bilayer to study the effects of GS on TCC-induced pore formation potency. I added GS stock to ensure only 100 or 200 nM GS concentration in the buffers of both chambers. The GS, up to 200 nM concentration, was tested and found independently unable to induce bilayer permeabilization; see supp. Figure 1 [9]. After about 20 min following the addition of GS (100 or 200 nM), I conducted the record of current traces across the lipid bilayer. At least three experimental repeats were made to demonstrate the 0, 100, and 200 nM GS effects on CD-induced ion pore currents across the lipid bilayer. 

For EP record experiments, we established a lipid bilayer workstation at our biophysics laboratory provided and installed by Warner Instruments LLC., Hamden, CT, USA. DIGIDATA 1440 (low-noise data acquisition system) and pCLAMP 10 CNS software for Windows were provided by Molecular Devices (Wokingham, United Kingdom) Ltd. We mimicked the EP experimental protocols [1,2]. Membrane current (in pA scale) was recorded at a filter frequency 20 kHz. Then, the current versus recorded time was plotted in Origin 9 (OriginLab Corp., Northampton, MA, USA).

### 2.2. Direct Detection Method

DDM was recently developed and applied to detect various lipid-bound MAAs in mole (M) fraction. We already addressed DDM for detecting CDs in vitro dioleoylphosphatidylcholine (DOPC) and aptamers in vitro DPPC and DPPS liposome systems using standard absorbance spectroscopy; for details, see refs. [39,40]. Existing techniques e.g., EP record of current, fluorescence measurements, etc., address the general lipid-binding effects, whereas the DDM helps detect the molecules directly at their target site(s). Let us denote B and UB for the solutions separated as a mole fraction of MAAs bound to lipids and unbound ones, respectively. Then, we use a NanoDrop (purchased from ThermoFisher Scientific, Waltham, MA, USA) or a Nanophotometer (purchased from Implen GmBH, Munich, Germany) to get the absorbance spectra that is specific for certain MAA. The wavelength (λ_MAA_) of the spectrum is actually MAA specific. λ_DNA_ = 260 nm is for DNA aptamers, λ_colchicine_ = 243 nm is for CD colchicine, etc. (please see the Sigma-Aldrich manual). Then, I perform the spectroscopy on both samples and quantify the concentrations of MAAs dissolved in both samples B and UB [39,40]. Using these detected concentrations, we calculate the molarities of both of the lipid-bound and lipid-unbound MAAs in the incubation tube. Then, these concentrations are normalized with the correct volume of the aqueous buffer in which the liposomes were formed; then, the liposomes were incubated with drugs before splitting the whole solution into B and UB. These lipid-bound drugs are nothing but considered as the DDM-detected liposome-bound drugs that are plotted later in this article. For DDM, I chose PC liposome, unlike the biological system mimicking lipid cocktail used in EP experiments, due to mainly the following two reasons: (i) to be consistent with membrane systems used in our earlier studies where DDM was first explained [39,40], and (ii) the molecular dynamics (MD) simulation results [2] hinted that in pairwise in silico kinetics, CD interaction with PC is relatively more deterministic than with PS. This confirmation was revealed considering the calculation of electrostatic and van der Waals interactions, thus proving the charge-based interactions to be the underlying molecular mechanisms behind the lipid interaction of CDs [2], hence their membrane adsorption [39,40]. Therefore, PC (only) liposome adsorption of CDs and the regulation of this adsorption mechanisms due to the GS effects on membrane physical properties may be better demonstrable.

## 3. Results

I have performed two sets of independent experimental studies. Firstly, I have performed the EP record of CD-induced membrane currents without and with the effects of GS on a bilayer membrane. Secondly, I addressed the quantitative membrane adsorption of CD molecules without and with the effects of GS using DDM. Both have been presented here in detail.

### 3.1. Electrophysiology Experiments Demonstrating GS Effects on TCC-Induced Lipid Bilayer Currents

**Figure 2:**
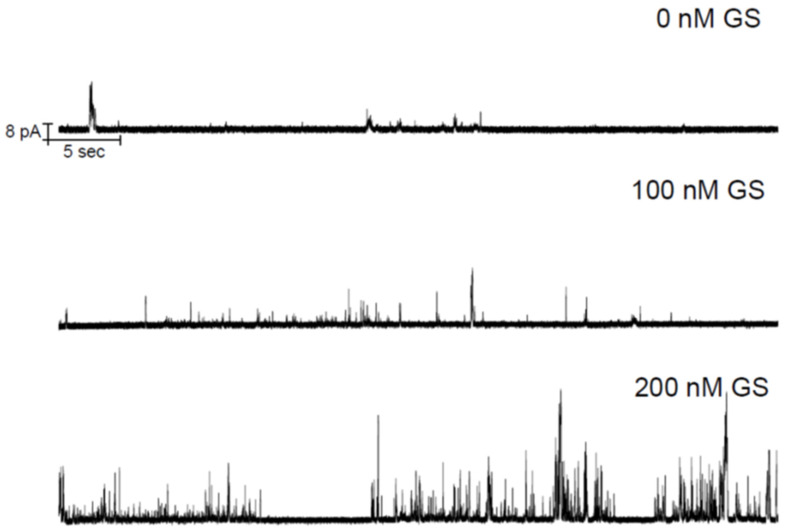
GS increases the chemotherapy drugs (CD)-induced ion pore induction potency. TCC (90 μM) alone permeabilizes lipid bilayer membranes by inducing nonzero current events (top panel). The middle and bottom panel represent the CD-induced current events being influenced by the effects of 100 nM and 200 nM GS, respectively, which are added into the aqueous phases in both chambers. As GS effects are added into the CD pore formation potency, the CD pore appearance frequency increases. So, we should not use too high CD concentrations to avert reaching at (or crossing over) the highest limit in the membrane current record, which is measurable in EP experiments. The traces were filtered at 20 kHz. POPE:POPS:POPC = 5:3:2, 500 mM NaCl + 90 μM TCC (*cis* side of the membrane), 500 mM NaCl + 0 μM TCC (*trans* side of the membrane), 100 mV, pH 7.4. 200 nM GS alone could not induce ion conductance across the membrane; see Figure S1 [9].

**Figure 3:**
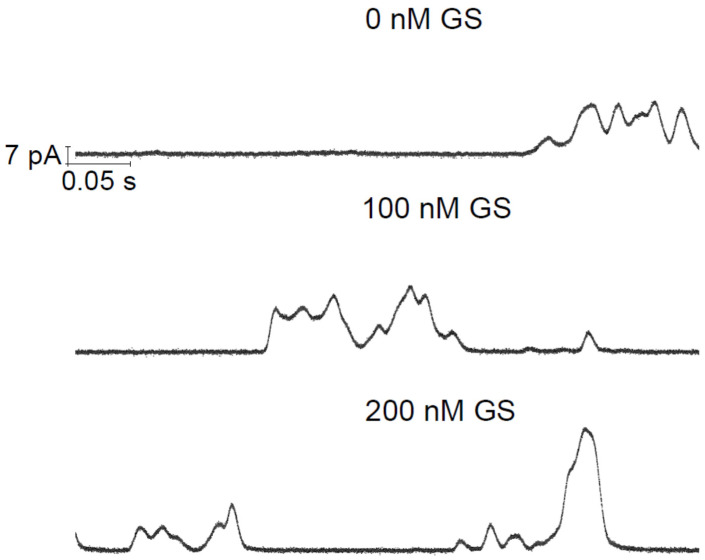
Short time (0.5 s) current traces: sections of Figure 2.

**Figure 4:**
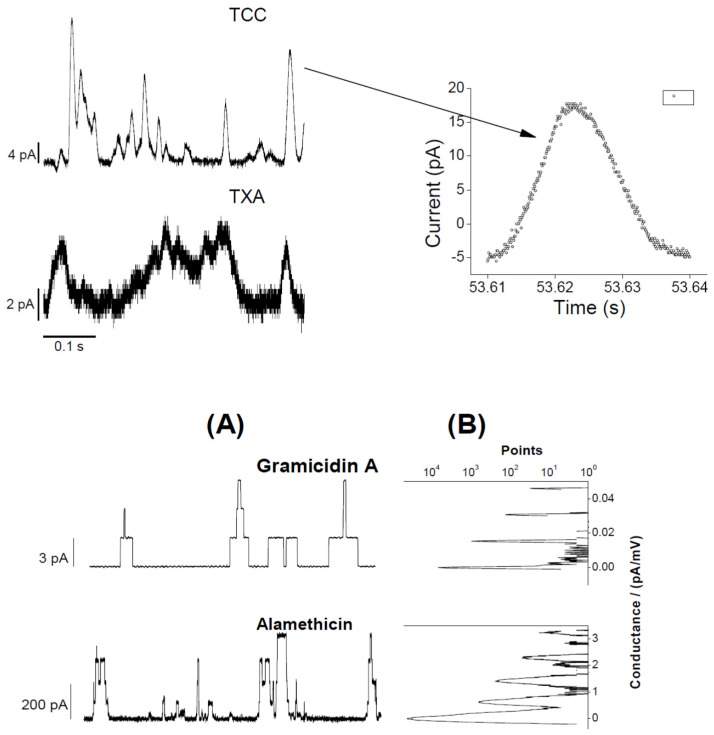
Comparison of two CD TCC and taxol (TXA) pore current traces with peptide (AgA (15) and alamethicim (Alm)) channel currents [9]. AgA (15) is a gramicidin A (gA) analogue having 15 amino acids in its sequence. The upper panel shows triangular-shape conductance events induced by TCC and TXA, both at 90 μM. pH = 5.7, *V* = 100 mV, filtered at 20 kHz. A single conductance event (pointed by a right arrow) shows all current points (in Origin 8.5 plot) and suggests for a triangular pattern, so does the pore current change spontaneously with time in both increasing and decreasing directions. The lower panel (**A**) illustrates rectangular-shape conductance events in gA and Alm channels (Ashrafuzzaman et al., 2008). gramicidin A (gA) channel activity was recorded at *V* = 200 mV and Alm at *V* = 150 mV and filtered at 2 and 20 kHz, respectively. Current values *I*_gA_ = 29 ± 2, 113 ± 5, 243 ± 9, and 386 ± 10 pA, respectively, are the discrete current levels 0, 1, 2, 3, …, etc. in the Alm channel. (**B**), the point count plots of the current traces through AgA (15) and Alm channels showing peaks at discrete values of conductance. The discreteness in conductance values for the CD pores is missing due to their triangular nature [1,2].

The EP-recorded lipid bilayer currents induced by two CD candidate molecules TCC and taxol were recently published [1,2]. Figure 2 demonstrates the GS effects on the TCC-induced pore currents across the lipid bilayer membrane. GS itself at considerably high concentrations is found, in our earlier studies, to induce defects in lipid bilayers [9]. GS permeabilizes the lipid bilayer membrane by creating instantaneous/transient conductance events. In current investigations, we have used GS concentrations that fall far below the minimum GS concentrations required for membrane permeabilization [9]. Therefore, the change in membrane current (through the appearance of an increased number of conductance events) (reflected in Figure 2, middle and bottom panels) over that of TCC-induced currents (Figure 2, top panel) is certainly not due to GS-induced current events. Moreover, GS was found not to induce pores but defects. In contrast, none of the recorded traces showed current events (Figure 3) that might resemble defects (transient current events), as seen in ref. [9]. Figure 3 presents the short time current traces showing triangular conductance events in all three conditions, not any of the traces resembling that for defects. This suggests that the conductance events observed here are all created due to TCC only. The triangular conductance events are toroidal type. This is concluded after comparing the single TCC pore current events with that of two other highly studied ion channels, gA and Alm channels (Figure 4).

In Figure 4, a comparison among patterns of CD and AMP pore currents has mainly been presented. Although AMP pore currents show clear pore stability and specific conductances, the CD pore current changes over the period of pore opening time. Huge fluctuations in CD pore current values are a hallmark of CD pores, unlike AMP pores having stable current levels. Moreover, the CD pore current level change is a time-dependent phenomenon, unlike AMP pore currents that experience instantaneous transitions between discrete current levels. 

**Figure 5:**
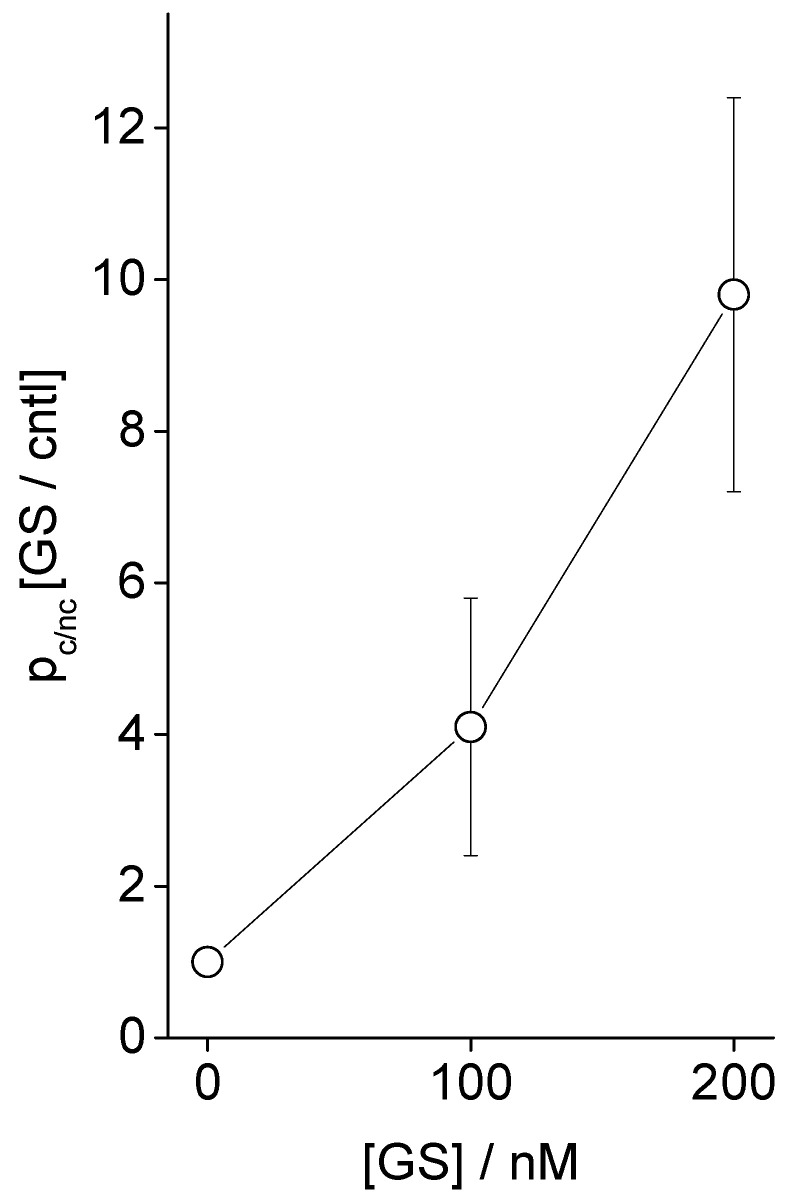
Effects of GS on CD-induced channel opening probability p_c/nc_ (Equation (1)). Along the y-axis is plotted the membrane conductance probability relative to the membrane nonconductance probability, being normalized by the value without the effects of GS (control condition). POPE:PS:PC = 5:3:2, 500 mM NaCl + 90 μM TCC (cis side of the membrane), 500 mM NaCl + 0 μM TCC (trans side of the membrane), 100 mV, pH 7.4. GS was added in the buffer on both sides of the bilayer.

We observe an increased appearance of conductance events as we increase the buffer incubating GS concentrations. Figure 5 shows that the membrane conductance probability (due to CD pore opening) over the membrane nonconductance probability increases with increasing GS concentrations in buffer. In Figure 5, along the y-axis is plotted the membrane conductance probability relative to the membrane nonconductance probability,

p_c/nc_= Σ_i_t_c,i_/t_nc_= Σ_i_t_c,i_/(Σ_i_t_c,i_+t_nc_)/(t_nc_/(Σ_i_t_c,i_+t_nc_)) = membrane conductance probability/membrane nonconductance probability. (1)

Here, t_c,i_ is the time (in seconds) during when the membrane conducts currents due to the opening of i-th CD pore, I = 1, 2, …, n. We consider that the membrane experiences the opening of n number of membrane conductance events due to the induction of CD pores over the duration of (EP recorded) time, t_rec_ (in second). The total time the membrane avoids conducting currents over the whole recorded time t_rec_ is t_nc_ = t_rec_ − Σ_i_t_c,i_. The calculation of t_c,i_ (proportional to the total points under peak) follows from the point count versus the conductance plot method explained in ref. [1,2] and shown in Figure 4 (see rightmost panels for gA and Alm channel data). The area under the peak at 0 pA/mV conductance of the membrane or 0 pA membrane current is nothing but the total point count proportional to the duration of the time when the membrane hosts no ion pores/channels. The sum of the areas under peaks at various nonzero conductance values (or for CD pores, sum of all points over all nonzero conductance values [1,2]) is the total point count proportional to the duration of time when the membrane conducts currents due to opening of the channels.

### 3.2. Liposome Binding of CDs Dissolved in Aqueous Phase-DDM to Detect Agents Directly at the Liposome

**Figure 6:**
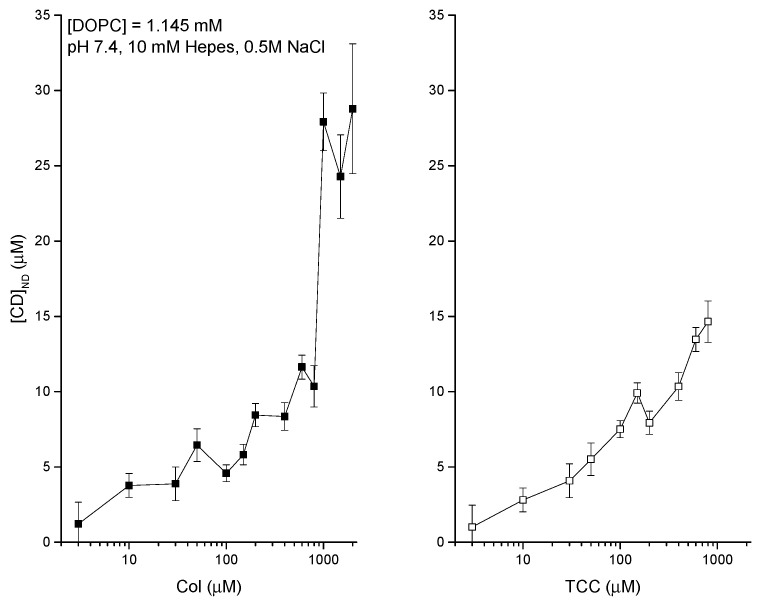
Two sets of data have been presented quantifying the direct detection method (DDM) detected liposome-bound colchicine for various aqueous environments bathing the liposome. Left panel: liposome-bound colchicine as a function of various Col concentrations in aqueous phase. Here, I tested up to reaching at the equilibrium condition on liposome adsorption of drugs. Right panel: liposome-bound TCC as a function of various TCC concentrations in aqueous phase. Here, I tested at low TCC concentrations and avoided reaching the equilibrium condition because of the insufficient amount of colchicine derivative TCC that could have been saved from our previous stock used in studies [1,2]. Concentration of lipids in the cuvette/buffer has been maintained for all investigations at [dioleoylphosphatidylcholine (DOPC)] = 1.145 mM.

**Figure 7:**
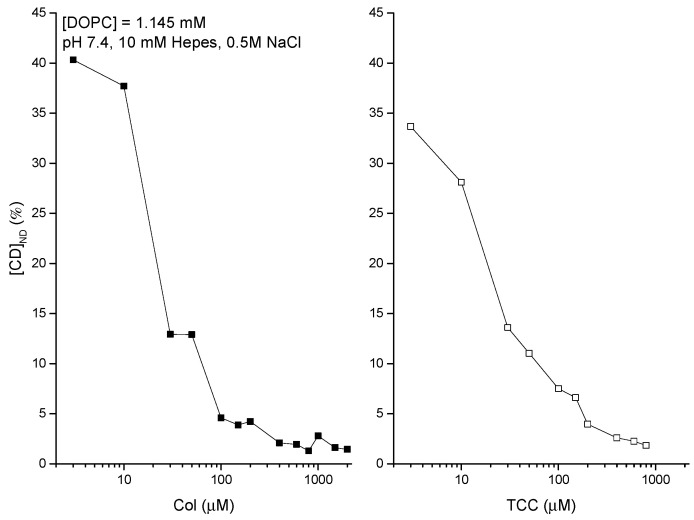
Percentage (%) of liposome-adsorbed CDs of their respective concentrations in buffer. These plots are deducted using the mean values presented in Figure 6.

The membrane adsorption of CDs is quantified in vitro liposome systems using DDM [39,40]. The adsorption phenomena get substantially regulated due to the environmental conditions of the aqueous phase bathing the liposomes. The experimental data are presented in Figure 6 (left and right panels), showing the liposome adsorption potency of both Col [39,40] and TCC against their various aqueous concentrations, bathing the liposomes. The DDM method allowed us to NanoDrop (ND) detect the actual CD concentrations inside liposomes (plotted along the y-axis). Figure 6 suggests another comparative aspect of liposome adsorption of CDs that as the buffer concentration of CDs increases, TCC starts showing modestly higher liposome adsorption potency reaching up to ≈30% higher TCC binding over Col binding to liposomes. As we deduct the percentage of CDs adsorbed in liposomes relative to their respective buffer concentrations (Figure 7), we observe that the binding potency decreases from around 40% at low CD concentration in buffer to as low as 1% at high CD concentration in buffer ≈1 μM, and it saturates beyond (tested for Col only). As the lipid concentration in the cuvette/buffer has been maintained for all investigations at lipid concentrations [DOPC] = 1.145 mM, we may deduct the drug/lipid molar ratios in the liposome, which fall up to less than the value ≈0.01 (for Col) and ≈0.013 (for TCC). The value of this ratio actually suggests how much drug molecules from the aqueous phase physically migrate into the lipid vicinity, and this process certainly has an important role of drugs in causing any effects on the membrane, especially those related to disrupting the membrane barrier properties.

**Figure 8:**
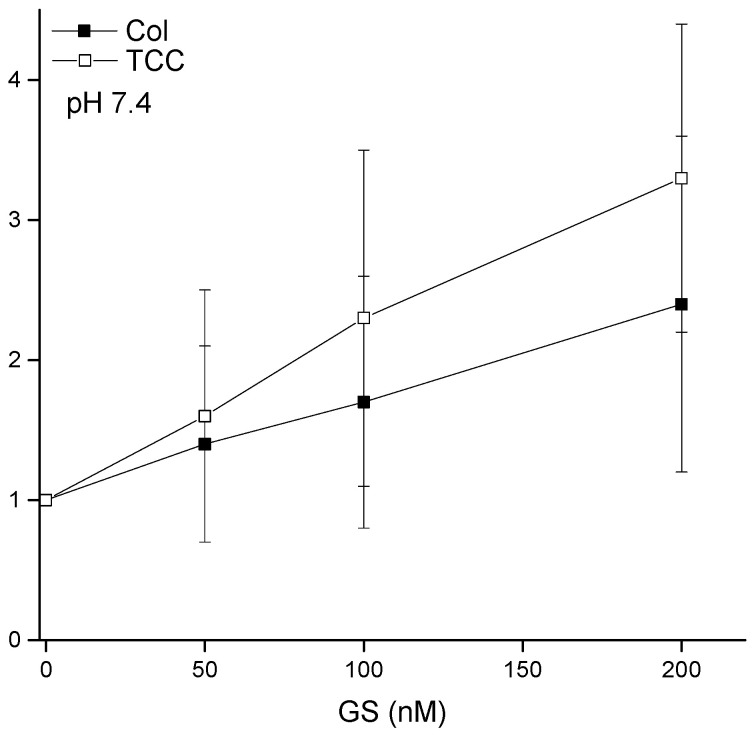
Liposome binding of CDs increases due to GS effects. The plots represent the liposome-bound CDs (normalized by the value of liposome-bound respective CD while the buffer was incubated with its 100 µM concentration) as a function of various concentrations of GS that coexisted with 100 µM CDs in the buffer. The concentration of lipids in the cuvette/buffer has been maintained for all investigations at [DOPC] = 1.145 mM.

After addressing the control experiments, I planned for investigating the effects of AMP GS in aqueous phase on the liposome adsorption of CDs. Our experimental plan was to observe the effects of GS at concentrations below the concentrations at which it disrupts membrane barrier properties regarding current conduction. Figure 8 shows the effects of GS on the liposome-binding potency of CDs, Col, and TCC, at their 100 μM concentrations. Results suggest that GS increases the liposome-binding potency of both Col and TCC. The relative CD-binding potency increases linearly with increasing GS concentrations. We observe modestly higher GS effects on TCC over Col binding to liposomes. CD molecules have huge solubility issues in aqueous buffer. Therefore, their membrane adsorption phenomena appear with huge statistical variations, as reflected in the large error bar at every data point in Figure 8.

### 3.3. pH Effects on Liposome Binding of CDs

**Figure 9:**
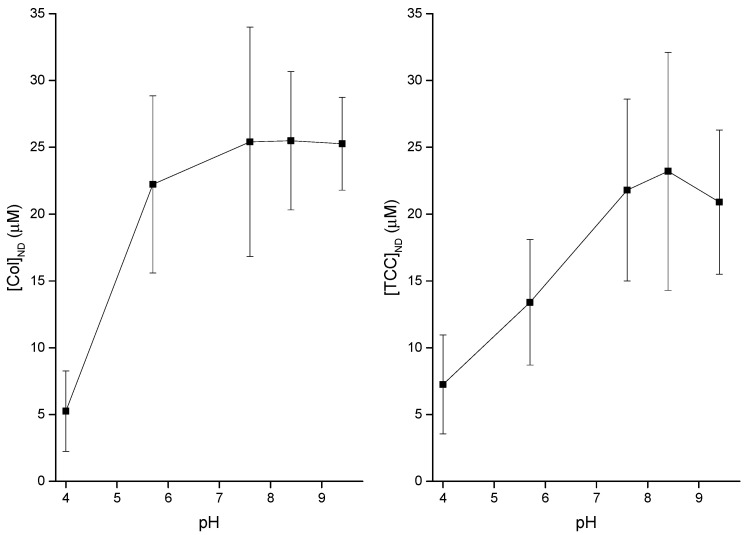
pH effects have been investigated on liposome binding of CDs, colchicine (Col) and TCC, while their concentrations in buffer are [Col] = 100 µM and [TCC] = 100 µM, respectively. Y-axis values are NanoDrop (ND) measured CD concentrations, which was detected directly at the liposomes using DDM. Different pH values have been obtained using the following conditions: pH 4, 5.7: 0.5 M NaCl + 20 mM acetate; pH 7.6, 8.4: 0.5 M NaCl + 20 mM Tris, pH 9.4: 0.5 M NaCl + 20 mM Glycine. The concentration of lipids in the cuvette/buffer has been maintained for all investigations at [DOPC] = 1.145 mM.

I demonstrated the buffer pH effects on the liposome adsorption potency of both CDs, Col and TCC. Figure 9 demonstrates the results. It is suggestive that the liposome adsorption of both Col and TCC increases with the increase of pH at a low pH value range (acidic condition). However, as the neutral condition is met, the alteration in pH condition loses any effects over a range of pH values, heading into the alkaline conditions. However, we must consider that in in vitro assays, we had to use various chemical compositions to achieve different pH conditions in the experimental buffer; see the caption of Figure 9. 

## 4. Discussion

Both TCC and GS are known, from independent EP experiments, to conduct lipid bilayer membranes. TCC is predicted to induce toroidal-type ion pores, while GS induces defects. The CD pores are found to be substantially stable, lasting over the varied duration of finite time, while the defects are transient types, lasting over no considerable duration of time. Both CD and GS require certain minimum concentrations to independently disrupt membrane electrical barrier properties. Below these minimum concentrations, either agent may not necessarily disrupt membrane barrier properties, but they may still exert other types of effects on the membrane physical properties. Recently, we found that GS increases the ion channel-forming potency of two other AMPs, gA and Alm [32]. The regulation of gA and/or Alm channel activity due to GS is found to follow identical behavior to those found from the investigated effects of Cpsn and TX100 on these channels [33,34,36,38]. GS increases CD (in the present study), gA, and Alm [32] pore-forming potency. Therefore, it is probably conclusive that GS has some common effects (yet unknown) on bilayer properties that regulate the membrane-hosted versatile ion channels. From a lot of studies, we already know that various MAAs, e.g. amphipathic molecules, antifusion peptides, amphiphiles, Cpsn, TX100, etc. alter bilayer physical properties (specifically reducing the bilayer stiffness), which regulate the integral channel functions [33,35,36,38]. Some of them promote opposite curvature profiles (e.g., GS and Cpsn promote negative curvature and TX100 promotes positive curvature) in lipid bilayers, but still, all these three are found to stabilize bilayer hosted channel functions irrespective of the differences in their structures [29,33,38]. Therefore, it may be predictable that GS does something similar (reduces stiffness) on the adsorbing lipid bilayer; as a result, the bilayer-hosted CD pores get stabilized or pore functions get promoted.

To go further dip inside, I performed experimental studies to address the GS effects on actual liposome adsorption of MAAs which (e.g., TCC) induce ion pores in lipid bilayers. EP-recorded membrane currents across the lipid bilayer doped with CD molecules clearly suggest that the membrane accommodates ion pore-forming agents inside, which is a key mechanism that is required to lead to compromising the membrane’s electrical insulation properties. This mechanism clearly requires substantial CD molecules to get adsorbed inside the membrane [1,2]. The membrane adsorption of CD molecules has been addressed here quantitatively using DDM [39,40]. There is a considerable concentration dependence on membrane adsorption of the pore-forming CD molecules observed. The DDM detection of CDs directly at the membrane has been found to get substantially influenced due to the membrane effects of GS at concentrations lower than that at which it instantaneously conducts the bilayer membrane. The increase in membrane adsorption of CDs due to GS indirectly suggests that GS perhaps exerts considerable effects on the potency of the membrane accumulation of MAAs. Thus, GS perhaps indirectly exerts effects on the pore formation of CDs inside lipid bilayers. This again confirms that GS perhaps alters bilayer physical properties in a similar manner to other amphiphile and amphipathic agents [33,35,38].

### 4.1. Modest Effects of pH on Both Membrane Adsorption of CDs and Induction of CD Pores

We reported in our earlier article that there was no considerable change in pore activity A observed over a range of physiologically relevant pH values between 5.7 and 8.5, see supp. Figure 2 [1,2]. Here, A = Σ_i_t_c,i_/t_rec_.

In comparable analysis of the buffer pH effects on CD adsorption and CD-induced pore formation in lipid membranes, both experimental observations show almost identical conclusions. Figure 9 shows modest pH effects in acidic condition for both Col and TCC, but as the buffer pH enters into neutral condition and beyond, we lose pH effects on CD adsorption in the lipid membrane. However, as mentioned earlier, these pH conditions in the aqueous phase have been maintained using varied chemical compositions, which may also exert additional chemical-specific effects that we cannot extract here. Therefore, we may not be able to make any bold statements using these pH effects data. Probably, further comparable studies on drugs’ cytotoxicity assays using cancer and normal biological cells may improve understanding (not covered in this article, but we are investigating this issue using various healthy and cancer cell lines). Cancer cells exist in acidic condition compared to the normal cells [43]. Acidification of the extracellular region (low pH ≈ 6.7–7.1) and concomitant alkalization of the intracellular cytoplasm (high pH ≈ 7.2) are among the hallmarks of cancer that lead to a reverse gradient in pH across cancer cell membrane versus that due to the corresponding values in normal cells,  ≈7.4 (extracellular region), and  ~ ≈ 7.2 (intracellular cytoplasm region), respectively [44]. So, cancer cell-targeted CDs may appear as very toxic for normal cells because a bit higher CD adsorption potency is observed at neutral condition relative to the acidic condition (Figure 9). Therefore, these CD candidates may appear with substantial off-target toxicity potency, which is one of the major concerns that many drug candidates need to tackle before appearing for clinical trials.

### 4.2. GS Effects on CD pore Formation and Liposome Adsorption Are Mutually Correlated

**Figure 10:**
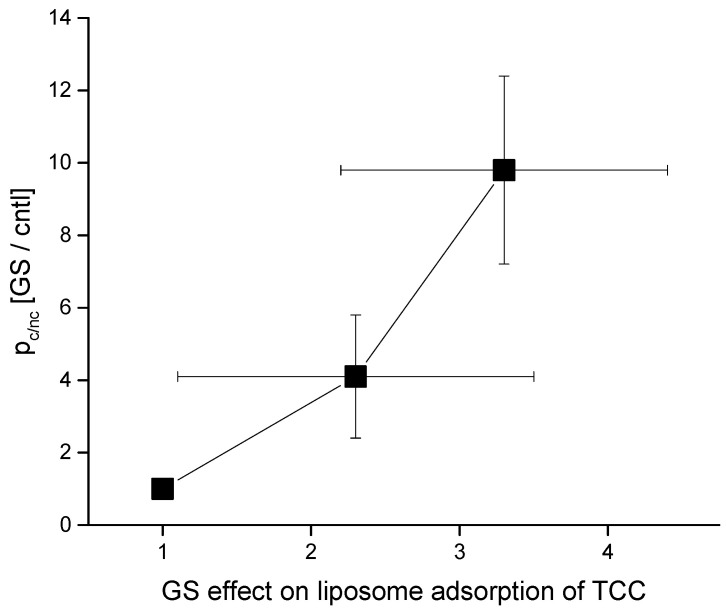
Correlation between GS effects in two different studies—GS effects on CD-induced membrane conductance versus liposome adsorption. Here, I have plotted the data for TCC only as we did not perform any electrophysiology record of membrane currents due to Col effects. Three data points represent the effects of [GS] = 0, 100, 200 nM

Figure 10 summarizes important universal aspects of GS effects. Both TCC-induced pore formation and its liposome adsorption draw effects from the GS presence in the aqueous phase, which are mutually correlated. Although as the GS concentration increases, the pore-formation potency tends to draw relatively higher effects than the liposome adsorption. We can not truly draw any hard conclusion on this matter, as we are limited within a low GS concentration range to avoid including especially any of its (high concentration-induced) own membrane conductance effects [9] with the CD-induced membrane conductance [1,2]. As we increased the GS effects on CD pore formation potency by adding high concentrations beyond 500 nM GS in the aqueous phase, we found the membrane conductance to reach an un-recordable high current range or infinity (tested, data not shown here).

## 5. Conclusions

We wish to suggest a few important findings. Firstly, an AMP that induces ion conduction events across a lipid bilayer may also alter bilayer physical properties at even concentrations which are not enough to produce effects that might break the bilayer electrical barrier properties. Thus, AMPs may also appear as bilayer physical property modulators (AMPs perhaps decrease membrane stiffness), which may also influence other AMPs’ or drugs’ effects on lipid bilayers including their pore-forming potency there. This hypothesis has been proved in this study, as we have found a negative curvature profile promoting AMP GS to upregulate the CD pore functions in the lipid bilayer. Both CD pore appearance frequency and stability were found to increase due to the GS effects on the CD pore-hosting bilayer. Secondly, the strong correlation between GS’s effects on membrane adsorption and the ion pore-forming potency of the CD molecules hints that the coexistence of various MAAs in the membrane vicinity (or even inside the membrane) may influence each other’s effects on the membrane, depending on their relative concentrations, in various ways, such as influencing the membrane adsorption, accumulation inside the membrane, formation of stable or transient structures, etc. Considering these results with other sets of studies [9,38] (some details are yet to be published) altogether certainly raises our general understanding of the universality of the effects of MAAs (independently or their binary combinations) on the physical properties of membranes. Thus, we may state that despite any peptide being unable to disrupt lipid bilayer electrical barrier properties, it may still exert effects on other types of bilayer physical properties such as the membrane curvature profile, bilayer stiffness, etc. that may subsequently regulate the effects of AMPs and general drugs on the cell membrane. This study will be considered important in understanding membrane-based drug effects (especially the CD effects); thus, we will provide feedback information to aid in designing novel drugs for various diseases in which the drugs need to interact with or cross through cell membranes and reach certain intracellular targets. 

## Data Availability

Data is contained within the article or supplementary material.

## Supplementary Materials

The following are available online at https://www.mdpi.com/article/10.3390/membranes11040247/s1, Figure S1. (**A**). GS effects on two types of gA channels, constructed by two gA monomers gA-(13) and AgA(15), respectively. a, Current trace recorded in the absence of gA with 200 nM GS added to both sides of a lipid bilayer. There is no evidence of GS channel activity. b and c, Current traces recorded from a bilayer that was doped with gA-(13) and AgA(15) in the absence (b) and presence (c) of 200 nM GS. GS increases gA channel activity. ii. GS alters Alm channel function. a and b, cur-rent traces of Alm channel activity before (a) and after (b) addition of 200 nM GS to both sides of the bilayer. The current transition amplitudes for gA-(13) and AgA(15) channels are 1.95 ± 0.12 pA and 3.05 ± 0.11 pA in the absence of GS, which did not change considerably due to the effects of GS. The applied transbilayer potential (V) was 200 mV. (**B**) GS effects on Alm channel activity. a. control Alm channel activity (0 μM GS) and b. Alm channel activity including the membrane effects of 200 nM GS, added to both sides of the bilayer. V = 150 mV. The electrophysiology records were made using identical strategies explained in refs. [9,35]. Lipid bilayer was constructed using 1,2-Dioleoyl-sn-Glycero-3-Phosphocholine/n-decane. Detailed analysis will appear in another manuscript, to be submitted by Ashrafuzzaman and Andersen; Figure S2: pH effects on CD-induced pore formation. A may be considered as the pore activity.
