# Supplementary material for: The Antimicrobial Peptide Gramicidin S Enhances Membrane Adsorption and Ion Pore Formation Potency of Chemotherapy Drugs in Lipid Bilayers
Source: Membranes (Basel). 2021 Mar 30;11(4):247. doi: 10.3390/membranes11040247 (PMC8067072; doi:10.3390/membranes11040247)
Supplement: Supplementary file 1 [file membranes-11-00247-s001.pdf]

membranes

Supplementary Materials: The antimicrobial peptide gramicidin S enhances membrane adsorption and ion pore formation potency of chemotherapy drugs in lipid bilayers

Md. Ashrafuzzaman

Biochemistry Department, Science College, King Saud University, Riyadh 11451, Saudi Arabia, mashrafuzzaman@ksu.edu.sa

Citation: Ashrafuzzaman. M. The Antimicrobial Peptide Gramicidin S Enhances Membrane Adsorption and Ion Pore Formation Potency of Chemotherapy Drugs in Lipid Bilayers. *Membranes* **2021**, *11*, x. https://doi.org/10.3390/xxxxx

Received: 07 January 2021
Accepted: 17 February 2021
Published: date

Publisher's Note: MDPI stays neutral with regard to jurisdictional claims in published maps and institutional affiliations.

Copyright: © 2021 by the authors. Submitted for possible open access publication under the terms and conditions of the Creative Commons Attribution (CC BY) license (http://creativecommons.org/licenses/by/4.0/).

A. GS effects on gA channels

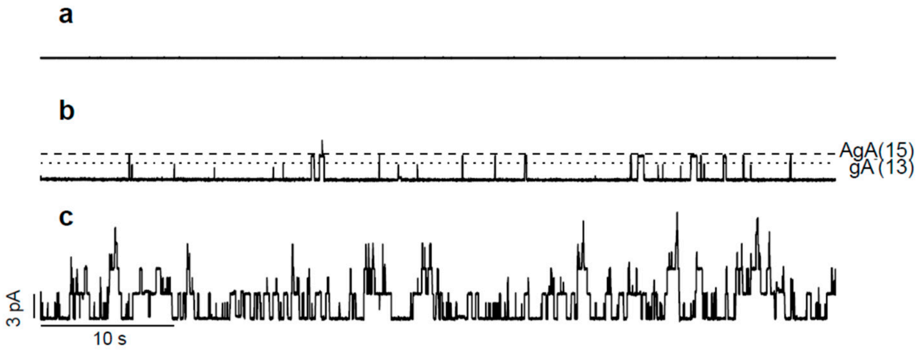

B. GS effects on Alm channels

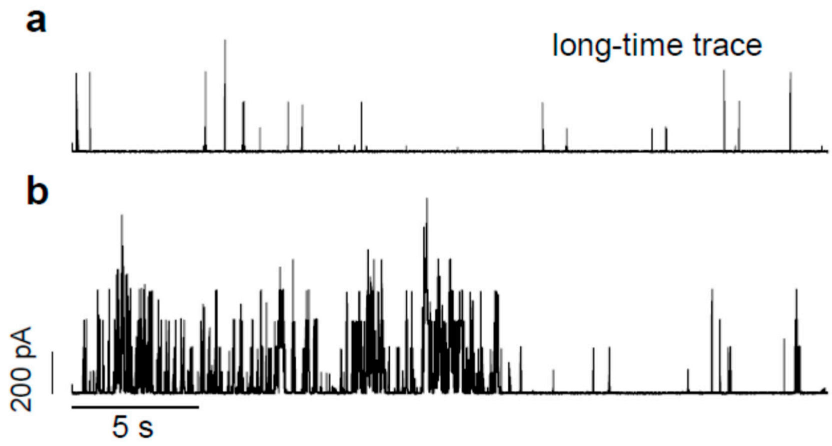

Figure S1. (**A**). GS effects on two types of gA channels, constructed by two gA monomers gA-(13) and AgA(15), respectively. a, Current trace recorded in the absence of gA with 200 nM GS added to both sides of a lipid bilayer. There is no evidence of GS channel activity. b and c, Current traces recorded from a bilayer that was doped with gA$^-$(13) and AgA(15) in the absence (b) and presence (c) of 200 nM GS. GS increases gA channel activity. ii. GS alters Alm channel function. a and b, current traces of Alm channel activity before (a) and after (b) addition of 200 nM GS to both sides of the bilayer. The current transition amplitudes for gA$^-$(13) and AgA(15) channels are 1.95 ± 0.12 pA and 3.05 ± 0.11 pA in the absence of GS, which did not change considerably due to the effects of GS. The applied transbilayer potential (V) was 200 mV. (**B**) GS effects on Alm channel activity. a. control Alm channel activity (0 μM GS) and b. Alm channel activity including the membrane effects of 200 nM GS, added to both sides of the bilayer. V=150 mV. The electrophysiology records were made using identical strategies explained in refs. [9,35]. Lipid bilayer was constructed using 1,2-Dioleoyl-sn-Glycero-3-Phosphocholine/n-decane. Detailed analysis will appear in another manuscript, to be submitted by Ashrafuzzaman and Andersen.

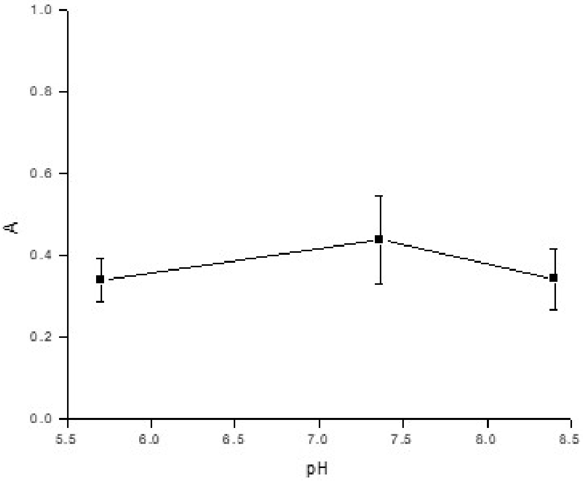

Figure S2. pH effects on CD-induced pore formation. A may be considered as the pore activity.
